# The evidence from in vitro primary fibroblasts and a randomized, double‐blind, placebo‐controlled clinical trial of tuna collagen peptides intake on skin health

**DOI:** 10.1111/jocd.16500

**Published:** 2024-07-29

**Authors:** Boontida Morakul, Veerawat Teeranachaideekul, Amaraporn Wongrakpanich, Jiraporn Leanpolchareanchai

**Affiliations:** ^1^ Department of Pharmacy, Faculty of Pharmacy Mahidol University Bangkok Thailand

**Keywords:** collagen peptides, density, elasticity, hydration, transepidermal water loss

## Abstract

**Background:**

Collagen peptides from various sources demonstrate benefits in health and well‐being both in vitro and in clinical trials. However, there is a scarce study of collagen peptides from Tuna on skin health.

**Aims:**

To investigate the impact of collagen peptides derived from Tuna (*Katsuwonus pelamis* and *Thunnus albacares*) on skin health, utilizing in vitro biological studies and a randomized controlled trial.

**Methods:**

In vitro biological studies on human dermal primary fibroblasts were evaluated in terms of collagen and elastin synthesis and senescent cell inhibition. A randomized, placebo‐controlled, double‐blind clinical trial was conducted on 72 women who were randomly assigned to receive either tuna collagen peptides (*n* = 36) or a placebo (*n* = 36) orally for 8 weeks and 2 weeks post‐ingestion by measuring skin hydration, transepidermal water loss (TEWL), skin elasticity, and skin density.

**Results:**

In vitro biological effects demonstrated dose‐dependent positive results in increasing collagen and elastin synthesis and reducing senescent cells. The effects on collagen and senescent cells plateaued at high concentrations. A clinical trial showed that the test group experienced a significant increase in skin hydration, elasticity, and density, along with a decrease in TEWL compared to the baseline. The test and placebo groups showed statistically significant differences at 8 weeks for all parameters except for the TEWL at the face. All positive effects were substantially retained even after 2 weeks of discontinuation.

**Conclusions:**

These findings demonstrate the significant potential of tuna collagen peptides to promote human skin health, warranting further investigation as a potential nutraceutical.

## 1 INTRODUCTION

Approximately 15% of the human total body weight is composed of skin. Skin physiology is significantly influenced by nutrition. Thus, good skin health indicates a person's well‐being and nutritional status. Various dietary supplements have been marketed with the promise of improving skin health and promoting a youthful appearance. Skin aging is the process by which the collagen density in the dermis diminishes. The dermal collagen network becomes progressively fractured, resulting in shorter and less organized fibers. Dermal fibroblasts produce fewer extracellular matrix components. The elastic fibers in the skin lose their structural integrity. Hyaluronic acid levels in the epidermis and dermis decline, which reduces the ability of the skin barrier to retain moisture, resulting in skin dryness.

Collagen is one of the main components of the skin providing strength and elasticity. Collagen peptides are the hydrolysates of collagen produced by enzymolysis of animal tissue, such as those from livestock, poultry, and fish. They are gaining interest as dietary supplements for their health and aesthetic benefits to the skin advantages over native collagen. Collagen peptides are effectively broken down into di and tripeptides that do not undergo additional intracellular hydrolysis before being transported across the intestinal mucosa by the transporter PEPT‐1.^1^ According to Kawaguchi's work, radioactively labeled collagen‐derived peptides could be absorbed and distributed throughout the rat skin tissue within 30 min of oral administration.^2^ The peptides could be retained at a high level in the rat skin for up to 14 days post‐administration.^3^ Previous studies have demonstrated that the oral ingestion of collagen peptides from various sources effectively improves stratum corneum (SC) water content,^4^ reduces transepidermal water loss (TEWL),^5^ and enhances skin elasticity.^6^, ^7^ However, there is still a lack of clinical evidence supporting the effectiveness of collagen peptides from tuna skin in promoting human skin health.

In this study, the collagen peptides derived from tuna skin (*Katsuwonus pelamis* and *Thunnus albacares*) were evaluated. The tuna collagen peptides gain attention over other sources according to the availability of food supply, environment safety via utilizing by‐products from the fish industry, and avoiding religious restrictions and health concerns associated with swine flu and bovine spongiform encephalopathy. The in vitro bioactivity effects of tuna collagen peptides were evaluated in terms of collagen and elastin synthesis and senescent cell inhibition against human dermal fibroblasts. Then, a randomized, placebo‐controlled, double‐blind trial was conducted to confirm the benefits on human skin. The trial involved 72 women who orally ingested tuna collagen peptides (TCP) for 8 weeks. The clinical benefits of this treatment were assessed by measuring skin hydration, TEWL, skin elasticity, and skin density at the face and forearm of volunteers. Additionally, the sustainability of the post‐ingestion effects was evaluated.

## 2 MATERIALS AND METHODS

### 2.1 Materials

The in vitro tests for studying biological effects were conducted using primary dermal fibroblasts human neonatal (HDFn) purchased from the American Type Culture Collection (ATCC^®^ PCS‐201‐010™), USA. Dulbecco's Modified Eagle's Medium (DMEM) and Modified Dulbecco's Phosphate Buffer Solution (DPBS) were supplied by Biowest, France. Fetal bovine serum (FBS) and antibiotic/antimycotic solutions were supplied from Gibco, USA. WST‐1 reagent was purchased from Roche Applied Science, Germany. L‐ascorbic acid and the Senescence Cells Histochemical Staining kit were purchased from Sigma‐Aldrich, USA. Human Collagen Type I ELISA was obtained from Cosmo Bio, USA. Elastin Assay‐Fastin™ Elastin kit was purchased from Biocolor, UK. The test sample in this study was TCP provided by Thai Union Ingredients Co., Ltd., Thailand, which was produced from the skin of wild‐caught tuna (*Katsuwonus pelamis* and *Thunnus albacares*) by the enzymatic treatment. In the study, TCP had an average molecular weight of less than 2000 Da with di, tripeptides representing more than 20% of the peptide content. The amino acid composition in 100 g of TCP is shown in the Table S1.

### 2.2 Study the biological effect of TCP on primary fibroblast

The HDFn (ATCC^®^ PCS‐201‐010™) were cultured in DMEM, supplemented with 10%v/v FBS and 1%v/v antibiotic/antimycotic solution. The cells were incubated at 37°C in a 5% CO_2_ environment.

#### 2.2.1 Cytotoxicity on primary fibroblast

The cytotoxicity effect of TCP on primary fibroblast was assayed by the WST‐1 method. Cell viability was determined based on the presence of succinate dehydrogenase enzyme in mitochondria, which can reduce 4‐[3‐(4‐Iodophenyl)‐2‐(4‐nitrophenyl)‐2H‐5‐tetrazolio]‐1,3‐benzene disulfonate (WST‐1) to the WST‐1 formazan. HDFn were seeded in a 96‐well plate at a density of 2 × 10^5^ cells/well, 24 h before the experiment. Cells were exposed to the TCP diluted with DMEM at various concentrations (1.95–500 mg/mL) for 24, 48, and 72 h. A 0.1%w/v of sodium dodecyl sulfate (SDS) solution and DMEM served as the positive and negative controls, respectively. After incubation, the sample was replaced with 100 μL of WST‐1 in DMEM (1:10). After 30 min of the incubation period, the absorbance of the samples was measured at 450 nm using a microplate reader (Infinite M Nano, Tecan, Austria). The percentage of cell viability was calculated using Equation (1). Concentrations of TCP yielding cell viability higher than 80% were chosen for further studies.

(1) $$\%\text{Cell viability}=\frac{\text{Absorbance of treated cells}}{\text{Absorbance of negative control cells}}\times 100$$

#### 2.2.2 Effect on collagen synthesis

In the experiment, HDFn were seeded in a 96‐well plate at a density of 2 × 10^5^ cells/well 24 h before the experiment. TCP sample was diluted with DMEM media to 15.62, 31.12, and 62.50 mg/mL. L‐ascorbic acid solution (30 μg/mL) and DMEM were used as the positive and negative controls, respectively. Cells were incubated with the sample in a 96‐well plate for 24 h. The culture supernatant was collected. The amount of collagen synthesized was determined by the Human Collagen Type I ELISA kit (Cosmo Bio Co., Ltd., USA) according to the manufacturer's instructions. The plate was pre‐coated with the human collagen type I. The biotinylated anti‐collagen antibody was then bound to both the pre‐coated human collagen type I and the collagen produced from the cells. The biotinylated anti‐collagen antibody was quantified by an HRP‐labeled avidin enzyme reaction. The absorbance was measured at 450 nm using a microplate reader.

#### 2.2.3 Effect on elastin synthesis

The elastin synthesis was determined using the Fastin elastin assay (Biocolor, UK). HDFn were seeded in a 96‐well plate at a density of 2 × 10^5^ cells/well 24 h before the experiment. TCP sample was diluted with DMEM to 15.62, 31.12, and 62.50 mg/mL. L‐ascorbic acid solution (30 μg/mL) and DMEM were used as the positive and negative controls, respectively. The prepared sample was incubated with the cells for 24 h. At the end of the experiment, the cells were removed from the well by trypsinization. The elastin was extracted from the cells using 0.25 M oxalic acid solution, precipitated, and collected using centrifugation at 10000 rpm, 4°C for 10 min. The precipitate was dyed with 1 mL of 5,10,15,20‐tetraphenyl‐21H,23H‐porphine tetrasulfonate (TPPS) solution. The excess dye was removed by centrifugation. The 250 μL of dye dissociation reagent was added to dissolve the elastin‐conjugated dye. The amount of elastin was then analyzed by a microplate reader at 513 nm.

#### 2.2.4 Effect on senescent cell inhibition

Senescence is a state of cell arrest that occurs as cells age. One way to identify senescent cells is to stain them with β‐galactosidase, an enzyme that is more abundant in the lysosomes of senescent cells than in normal cells. Positive staining of β‐galactosidase, which appears blue, indicates cell senescence.

The experiment was done using the Senescence Cells Histochemical Staining kit (Sigma‐Aldrich, USA). The cells were seeded 24 h before the treatment in a 96‐well plate at a concentration of 2 × 10^5^ cells/well. TCP sample was diluted with DMEM to the final concentrations of 15.62, 31.12, and 62.50 mg/mL. The diluted TCP sample was added to each well and incubated for 48 h. The cells incubated with blank DMEM were used as the control. The cells were subcultured for three passages every 48 h. At the end of the experiment, the cell culture media was discarded. Cells were rinsed with 1 mL of DPBS twice, fixed with the fixation buffer for 6 min, rinsed with DPBS, and stained with the staining mixture. The plate was incubated at 37°C for 12 h. The number of senescent cells stained in blue was counted using an inverted microscope (ZEISS Axio Vert.A1, Carl Zeiss Microscopy, USA). The percentage of senescent cell inhibition was calculated compared to the control, as shown in Equation (2).

(2) $$\%\text{Inhibition senescence cells}=\frac{\left(\mathrm{No}.\;\text{of senescence cells of control}-\mathrm{No}.\;\text{of senescence cells of sample}\right)}{\mathrm{No}.\;\text{of senescence cells of control}}\times 100$$

### 2.3 Clinical study

#### 2.3.1 Test product and placebo

In the clinical study, the test product containing TCP and the placebo were prepared as a food supplement in powder form. The test product was a blend of 99.5%w/w TCP and 0.5%w/w flavoring agent (blackcurrant flavor). The placebo was composed of 99.5%w/w maltodextrin, 0.5%w/w flavoring agent (blackcurrant flavor), and the coloring agents (tartrazine and sunset yellow) in sufficient amounts to imitate the color of the test product. Maltodextrin, a polysaccharide food additive, is widely used as an oral placebo in clinical trials due to its highly soluble in water. As a food additive, maltodextrin is employed to improve the texture, taste, and stability of food product formulation.^8^, ^9^ The test product and placebo were orally taken at 5 g daily after being dissolved in water. The selected dose in this study falls within the effective range of 2.5–12 g for hydrolyzed collagen supplementation on skin aging, as determined by the systematic review of de Miranda et al.^10^

#### 2.3.2 Study design and ethical aspects

This study was a randomized, double‐blind, placebo‐controlled clinical trial conducted following the International Conference on Harmonization in Good Clinical Practices (ICH‐CGP) principles and the Declaration of Helsinki. All participants in this study were voluntary and provided written informed consent after a full explanation of the risks and benefits of the procedure.

#### 2.3.3 Study participants

Prior to conducting the clinical research, the sample size calculation was based on the previous clinical study on the relationship between collagen and skin attributes by Bolke et al.^11^ According to the comparison of two means, with an estimated 80% power, and 5% significance, 33 participants per group were required. Considering the various exclusion conditions (10%), we finally included 36 participants in each group. Therefore, the total number of participants involved in the study was 72. The subjects were healthy women aged 40–60 who met the criteria as follows: (1) no smoking; (2) no wounds, scratches, tattoos, or skin disease in the test area; (3) no history of allergies to the ingredients in dietary supplements such as collagen and marine fish; (4) no receiving procedures such as injection of botulinum toxin, filler, collagen, laser treatment at least for 3 months, and thread lifting at least for 12 months before the experiment and throughout the experimental period; (5) no use of oral and topical drugs and skincare that might affect the results, such as steroids, antihistamine drug, hydroquinone, and vitamin A derivative; (6) no starting or changing the hormonal drugs containing estrogen or progesterone at least for 1 month before the experiment and throughout the experimental period; (7) no pregnancy or lactation or planned pregnancy during the study period; (8) agreeing to participate in the clinical trial. In total, 72 participants who met all inclusion criteria were included in the study.

#### 2.3.4 Study procedure

Eligible participants were assigned a randomization number by a blinded investigator. The randomization code was assigned to the participants according to the ordering of their screening visit. The random groups were created using Microsoft Excel. Equal numbers of participants were randomly assigned to the TCP and placebo groups. The TCP and placebo were sealed in sachets that were identical in appearance and labeled per the ICH‐GCP requirements. The group allocations were coded and concealed from the participants, investigators, and statistician throughout the study. The code was disclosed after all primary statistical analyzes were completed. The duration of the study was 10 weeks. All subjects consumed the assigned product once daily for 8 weeks. At the time before the first intake of the assigned product (T0), after 4 (T1) and 8 (T2) weeks of intake, and after a follow‐up period (without intake) of 2 weeks (T3), subjects were dermatologically evaluated.

#### 2.3.5 Compliance

The product compliance was assessed by counting the returned unused products at week 4 and week 8 and determined by the number of dosage units taken divided by the number expected, multiplied by 100.

#### 2.3.6 Skin health evaluation

The measurement of efficacy on skin health was performed. The face (cheek) and forearm were selected as the test areas to evaluate skin hydration, TEWL, skin elasticity, and skin density. The measurement areas remained the same throughout the study. All experiments were conducted under a controlled and stable environment (temperature 23 ± 2°C and humidity 40%–60%RH) and the subjects were given 15–30 min to acclimatize before undergoing skin measurements.

##### Skin hydration

Skin hydration was measured using a MoistureMeter SC (Delfin Technologies, Finland), a non‐invasive device that measures the skin's dielectric constant and converts it into skin water content. Higher measured values indicate greater skin hydration. At least three measurements were taken in each test area at each time point.

##### Transpeidermal water loss (TEWL)

TEWL was measured using a VapoMeter (Delfin Technologies, Finland), which harbors a humidity sensor in a probe chamber that records changes in relative humidity inside the chamber during the measurement and automatically calculates TEWL in g/m^2^/h. A decrease in the measured TEWL value might suggest an improvement in the skin barrier function and a reduction in water loss. Analysis was performed using the average of three values.

##### Skin elasticity

The skin elasticity was measured using the Dermalab^®^ Combo elasticity probe (Cortex Technology ApS, Denmark). The viscoelasticity (VE) values were evaluated on a selected test area with a setting for a normal skin condition (400 mbar negative pressure). Each measurement was repeated three times. The VE value, represented in MPa, is the ratio of elastic recovery to the total deformation and represents biological elasticity. Higher values of VE correspond to better skin elasticity.

##### Skin density

The skin density was evaluated using ultrasonography with Dermalab^®^ Combo, 20 MHz ultrasound probe (Cortex Technology ApS, Denmark). A constant gain curve was applied for each subject, and the ultrasonographic image was visualized and recorded. The digital image analysis was analyzed by the SkinLab software version 2.1.1.7 (Cortex Technology ApS, Denmark). The epidermis was identified by a hyper‐reflecting band. The sub‐epidermal hypo‐ and the hyper‐echogenic bands, which correspond to the papillary and reticular dermis, respectively, were chosen on the screen, and the dermis density was determined on the entire skin block. The dermis density is measured as intensity. The measurements were performed three times, and the average was calculated.

### 2.4 Adverse events

The safety and tolerability of the administration of treatment were evaluated at week 4, week 8, and week 10. The investigators asked the subjects whether they had gastrointestinal discomfort, body skin tingling, itching, and other symptoms. During the whole study, the participants could inform the investigators of any adverse events immediately once they experienced any uncomfortable feelings. The adverse events were documented in the study record and classified based on the description, duration, intensity, frequency, and outcome. The investigators assessed all adverse events and determined casualty and intensity.

### 2.5 Statistical analysis

Statistical analysis was conducted using GraphPad PRISM version 10.0.0 (GraphPad Software, La Jolla, CA, USA). The difference in the in vitro biological results was analyzed using a one‐way ANOVA by Tukey's honestly for multiple comparisons. The clinical study on skin parameters, including skin hydration, TEWL, elasticity, and density, was evaluated at day 0 (T0), week 4 (T1), week 8 (T2), and week 10 (T3). Efficacy was determined by relative changes of these parameters, which were determined by the following differences in the means: T1‐T0, T2–T0, and T3–T0. The difference in the mean of skin parameters within and between groups was analyzed using a two‐way repeated measures ANOVA with Tukey's multiple comparisons test. The primary outcomes and test hypotheses of the trial were based on the comparison of the TCP group and placebo group regarding the relative change (T2–T0) of (i) skin hydration, (ii) TEWL, (iii) skin elasticity, and (iv) skin density. A *p‐*value of less than 0.05 was considered significant.

## 3 RESULTS AND DISCUSSION

### 3.1 Biological effect on primary fibroblast

#### 3.1.1 Cytotoxicity in primary fibroblast

**FIGURE 1:**
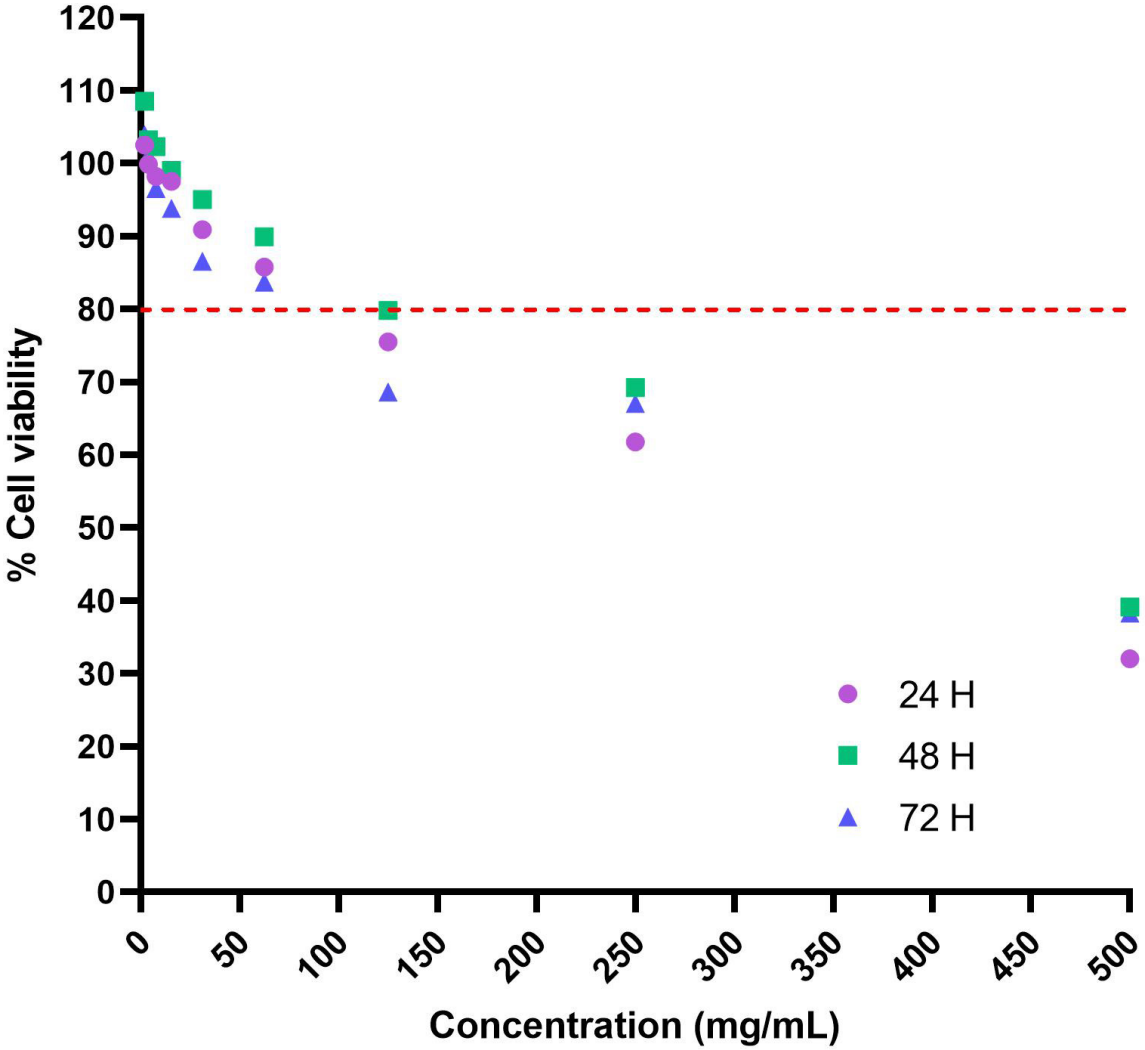
Relative cell viability (%) of HDFn primary fibroblast after exposure to TCP for 24 (purple), 48 (green), and 72 (blue) h. The PBS‐treated group was considered to have 100% cell viability. The data were plotted according to TCP concentration (mg/mL) and expressed as mean ± SD (*n* = 3).

To assess the cytotoxicity of TCP, HDFn primary fibroblasts were exposed to a range of concentrations (1.95–500 mg/mL) for 24, 48, and 72 h (Figure 1). The results showed that TCP cytotoxicity was concentration‐dependent, with cell viability remaining above 80% at concentrations of 62.50 mg/mL or less. These concentrations of TCP demonstrated low or no cytotoxicity to HDFn primary fibroblasts, making them suitable for subsequent biological effect studies. Therefore, TCP concentrations of 15.62, 31.12, and 62.50 mg/mL were selected for subsequent biological effect studies.

#### 3.1.2 Effect on collagen synthesis

**FIGURE 2:**
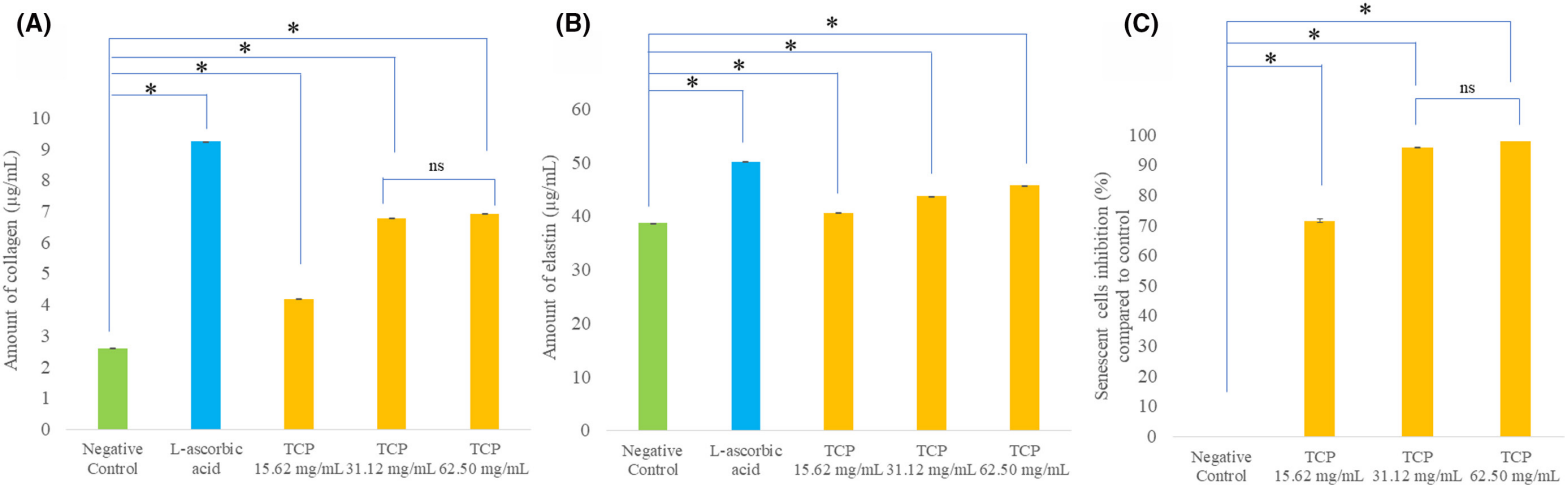
Amount of collagen (μg/mL) (A), amount of elastin (μg/mL) (B), and senescent cells inhibition (%) compared to control (C) of the negative control, L‐ascorbic acid, and TCP at concentrations of 15.62, 31.12, and 62.50 mg/mL, tested in HDFn primary fibroblast. The data were expressed as mean ± SD (*n* = 3). *indicates the significant difference at *p* < 0.05 compared to the negative control. “ns” means no significant difference between those samples.

To evaluate the effect of TCP on collagen synthesis, HDFn primary fibroblasts were incubated with TCP at various concentrations (15.62, 31.12, and 62.50 mg/mL) for 24 h. The collagen levels were then measured using the Human Collagen Type I ELISA kit. As shown in Table 1 and Figure 2A, cells treated with all tested TCP concentrations showed a significant increase in collagen levels compared to the negative control (*p* < 0.05). Simultaneously, the positive control (L‐ascorbic acid) also demonstrated a significant increase in collagen levels. No significant difference in collagen synthesis was observed between cells treated with 31.12 and 62.50 mg/mL of TCP (*p* > 0.05). This result suggests that TCP may enhance collagen synthesis in a dose‐dependent manner; however, this effect may plateau at higher concentrations. The results obtained were in alignment with the findings of Lee et al., which observed an increased mRNA expression of collagen 1 and procollagen type 1 peptide levels in human dermal fibroblasts when treated with fish skin collagen tripeptides, compared to a control group.^12^ Similarly, Offengenden et al. demonstrated that chicken collagen hydrolysates can stimulate type 1 collagen synthesis and cellular proliferation in human dermal fibroblasts. This effect depends on both the molecular weight and the specific action of a particular peptide.^13^

**TABLE 1:** Effect of TCP on collagen synthesis, elastin synthesis, and senescent cell inhibition compared with the negative control (DMEM) and the positive control (0.03 mg/mL of L‐ascorbic acid).

Sample group	Concentration (mg/mL)	Effect on collagen synthesis		Effect on elastin synthesis		Senescent cells inhibition		
		Amount of collagen (μg/mL)	Collagen increased (%)	Amount of elastin (μg/mL)	Elastin increased (%)	Amount of non‐senescent cells (%)	Amount of senescent cells (%)	Senescent cells inhibition (%) compared to control
Negative control	‐	2.61 ± 0.01^d^	‐	38.71 ± 0.00^e^	‐	68.81 ± 2.11^c^	31.33 ± 2.11^a^	0.00 ± 0.00^c^
Positive control (L‐ascorbic acid)	0.03	9.25 ± 0.00^a^	254.48 ± 0.72^a^	50.22 ± 0.00^a^	29.73 ± 0.12^a^	‐	‐	‐
TCP	15.62	4.20 ± 0.00^c^	60.99 ± 1.09^c^	40.63 ± 0.00^d^	4.97 ± 0.14^d^	91.10 ± 2.71^b^	8.89 ± 2.71^b^	71.48 ± 0.13^b^
	31.12	6.79 ± 0.00^b^	160.18 ± 4.15^b^	43.72 ± 0.00^c^	12.96 ± 0.06^c^	98.71 ± 1.24^a^	1.28 ± 1.24^c^	95.89 ± 0.60^a^
	62.50	6.93 ± 0.00^b^	165.52 ± 1.88^b^	45.75 ± 0.00^b^	18.20 ± 0.04^b^	99.37 ± 0.43^a^	0.62 ± 0.43^c^	98.01 ± 0.15^a^

*Note*: Data are expressed as mean ± SD (*n* = 3). The different letter labeled indicates the significant difference of the sample from each other sample in the same considered variable (same table's column) with a 95% confidence interval by Tukey's Honestly significant different test.

#### 3.1.3 Effect on elastin synthesis

The determination of the elastin synthesis of TCP on HDFn primary fibroblasts compared to the negative control (DMEM) and the positive control (L‐ascorbic acid) is presented in Table 1 and Figure 2B. The positive control exhibited a significant increase in the elastin levels by 29.73%. In the meantime, as the concentration of TCP increased, there was a significant rise in elastin synthesis by 18.20%, 12.96%, and 4.97% for concentrations of 62.50, 31.12, and 15.62 mg/mL, respectively (*p* < 0.05). The result suggests that TCP can promote elastin synthesis, with the effect being more pronounced at higher concentrations. The findings were consistent with the study conducted by Shiratsuchi et al., which demonstrated that when human skin fibroblasts were treated with elastin hydrolysate and dipeptides “Prolyl‐Glycine,” there was a noticeable increase in both cell proliferation and elastin synthesis.^14^ Additionally, the previous report by Edgar et al. showed that collagen peptides (ranging from 0.3–8 kDa) significantly increased fibroblast proliferation and elastin synthesis, while also inhibiting the release of the matrix metalloproteinase‐1 (MMP‐1) and MMP‐3, leading to a reduction in elastin degradation in primary human dermal fibroblasts.^15^

#### 3.1.4 Effect on senescent cell inhibition

The effect on senescent cell inhibition was evaluated by measuring the β‐galactosidase activity, visualized as blue‐stained cells (Supplementary S2). The TCP treatment significantly reduced the percentage of senescent cells compared to the control group (*p* < 0.05) (Table 1 and Figure 2C). A similar finding by Lee et al. demonstrated that collagen peptides contributed to the prevention of cell aging. Specifically, the administration of collagen tripeptide from fish skin was shown to improve the accumulation of advanced glycated end products, reduce the production of denatured collagen production, and decrease reactive oxygen species in dermal fibroblasts.^13^ In addition, the result also aligns with the study by Chae et al. showing the ability of collagen peptides derived from the skin of the golden threadfin bream (*Nemipterus virgatus*) to prevent collagen type I inhibition mediated by cortisol in senescent HDFs and reconstituted human skin models.^16^ In this study, the effect of TCP on senescent cell inhibition was dose‐dependent. However, there was no significant difference in the percentage of senescent cell inhibition between the TCP concentrations of 31.12 and 62.50 mg/mL (*p* > 0.05). TCP exhibits a dose‐dependent effect on senescent cell inhibition but reaches a plateau at a certain concentration, similar to its effect on collagen synthesis.

The results from the in vitro biological activities of TCP in this study highlighted the potential of collagen peptides in combating aging and enhancing skin health. It could serve as a preliminary screening to evaluate the potential anti‐aging effects of TCP on the skin in a clinical context. Consequently, a follow‐up clinical trial involving human participants was initiated to further validate these initial findings.

### 3.2 Study participants

**FIGURE 3:**
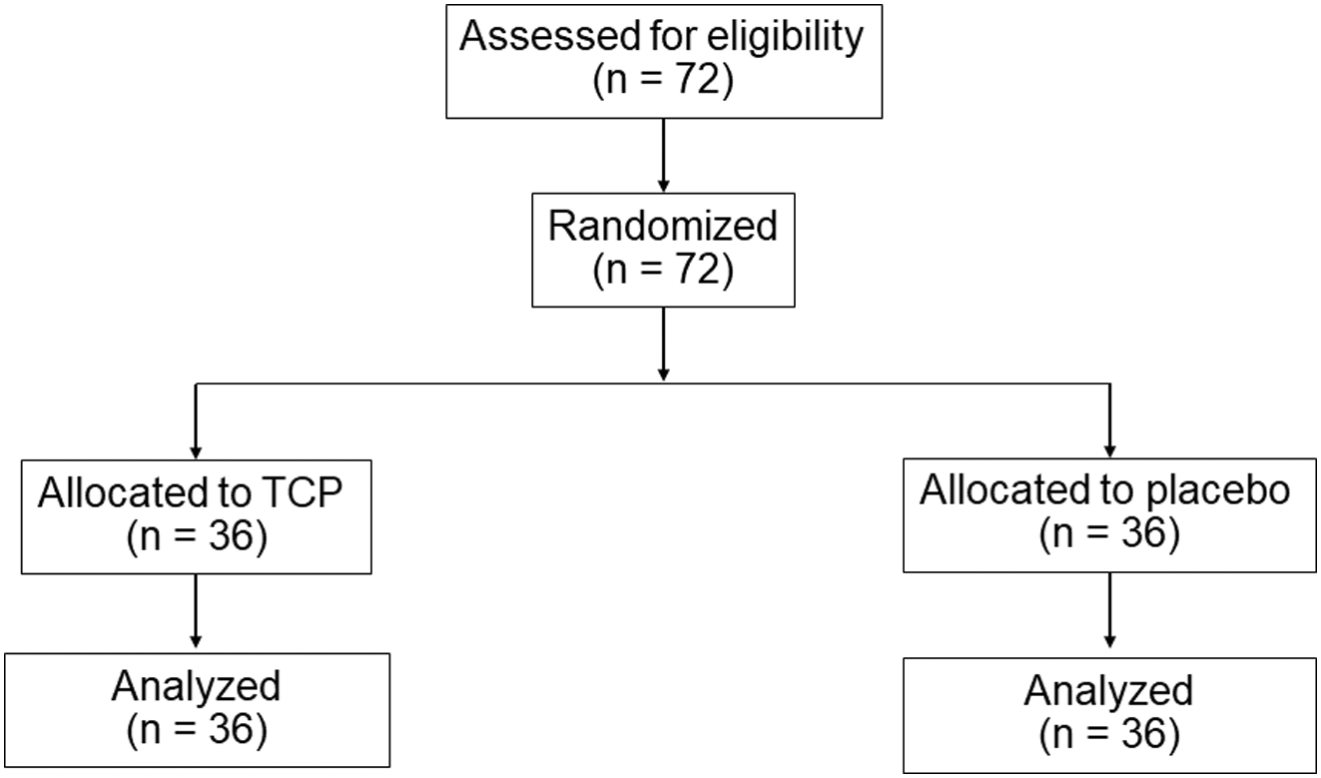
Flowchart diagram of the clinical study.

In this study, participants (*n* = 72) were randomized at the outset and divided equally into the TCP group (*n* = 36) and the placebo group (*n* = 36). Each participant was administered 5 g of either TCP or placebo orally daily for a duration of 8 weeks, followed by a 2‐week discontinuation of the test products. All 72 participants successfully completed the study as per the protocol (Figure 3). There were no differences between the TCP and placebo groups regarding sex, age, and parameter‐related initial skin condition (Table 2). Notably, throughout the study duration, no side effects or adverse events were reported.

**TABLE 2:** Skin hydration, TEWL, VE, and intensity at the face and forearm of the healthy volunteers before and after treatment with TCP and placebo for 4 and 8 weeks, and after discontinuing use for 2 weeks (at 10 weeks).

Parameter	Position	Time	Product				*p*‐value^a^
			TCP (*n* = 36)	*p*‐value^b^	Placebo (*n* = 36)	*p*‐value^b^	
Sex			Female (*n* = 36)		Female (*n* = 36)		‐
Age (years)			48.8 ± 5.0		48.9 ± 4.9		‐
Skin hydration (A.U.)	Face	Initial	47.6 ± 2.9	‐	46.4 ± 3.1	‐	0.7667
		4 weeks	62.8 ± 2.4	<0.0001***	48.9 ± 2.8	0.5670	0.0003***
		8 weeks	72.6 ± 2.8	<0.0001***	45.4 ± 2.6	0.8430	<0.0001***
		10 weeks	69.9 ± 2.7	<0.0001***	43.3 ± 2.5	<0.0001***	‐
	Forearm	Initial	26.4 ± 1.5	‐	23.6 ± 1.2	‐	0.1702
		4 weeks	28.7 ± 1.4	0.0351*	25.3 ± 1.4	0.1428	0.1024
		8 weeks	33.1 ± 1.8	<0.0001***	24.3 ± 1.2	0.7316	<0.0001***
		10 weeks	31.7 ± 1.8	0.0002***	23.6 ± 1.1	0.9636	‐
TEWL (g/m^2^/h)	Face	Initial	13.4 ± 0.6	‐	12.9 ± 0.5	‐	0.5688
		4 weeks	12.7 ± 0.5	0.3820	12.7 ± 0.5	0.7831	0.9109
		8 weeks	11.7 ± 0.4	0.0011**	12.7 ± 0.4	0.7889	0.1027
		10 weeks	11.7 ± 0.4	<0.0001***	12.5 ± 0.4	0.2417	‐
	Forearm	Initial	9.8 ± 0.4	‐	9.1 ± 0.3	‐	0.1518
		4 weeks	9.4 ± 0.3	0.2388	9.5 ± 0.3	0.3270	0.8986
		8 weeks	9.1 ± 0.3	0.0191*	9.9 ± 0.3	0.0071**	0.0445*
		10 weeks	9.3 ± 0.2	0.0396*	9.9 ± 0.3	0.0015**	‐
VE (MPa)	Face	Initial	4.8 ± 0.2	‐	4.6 ± 0.3	‐	0.4588
		4 weeks	4.9 ± 0.2	0.6848	4.2 ± 0.2	0.0618	0.0169*
		8 weeks	5.2 ± 0.2	0.0116*	3.9 ± 0.2	0.0002***	<0.0001***
		10 weeks	5.1 ± 0.2	<0.0001***	3.9 ± 0.2	<0.0001***	‐
	Forearm	Initial	6.3 ± 0.3	‐	5.8 ± 0.2	‐	0.0949
		4 weeks	6.3 ± 0.2	0.9796	5.8 ± 0.2	0.9835	0.1313
		8 weeks	6.9 ± 0.2	0.0005***	5.5 ± 0.2	0.1105	<0.0001***
		10 weeks	6.8 ± 0.2	0.0042**	5.4 ± 0.2	0.0143*	‐
Intensity (A.U.)	Face	Initial	44.4 ± 1.9	‐	47.1 ± 1.8	‐	0.3010
		4 weeks	50.2 ± 2.1	<0.0001***	50.3 ± 1.7	0.0155*	0.9917
		8 weeks	53.6 ± 1.8	<0.0001***	47.0 ± 1.5	0.9978	0.0107*
		10 weeks	51.0 ± 1.6	<0.0001***	45.7 ± 1.5	0.2611	‐
	Forearm	Initial	44.0 ± 1.5	‐	45.1 ± 1.4	‐	0.5619
		4 weeks	47.2 ± 1.4	0.0036**	45.2 ± 1.6	0.9987	0.3175
		8 weeks	50.2 ± 1.2	<0.0001***	42.7 ± 1.3	0.0338*	0.0002***
		10 weeks	47.3 ± 1.2	0.0065**	41.9 ± 1.4	0.0022**	‐

*Note*: The experiments were performed in triplicate. Data are expressed as mean ± SEM (*n* = 36 per group).

^a^ *p*‐value is the comparison of the TCP with the placebo at the same time and position.

^b^ *p*‐value is the comparison of the same product with its initial time at a similar position.

* Indicates the significant difference at *p* < 0.05.

** Indicates the significant difference at *p* < 0.01.

*** Indicates the significant difference at *p* < 0.001.

### 3.3 Effect on skin hydration

**FIGURE 4:**
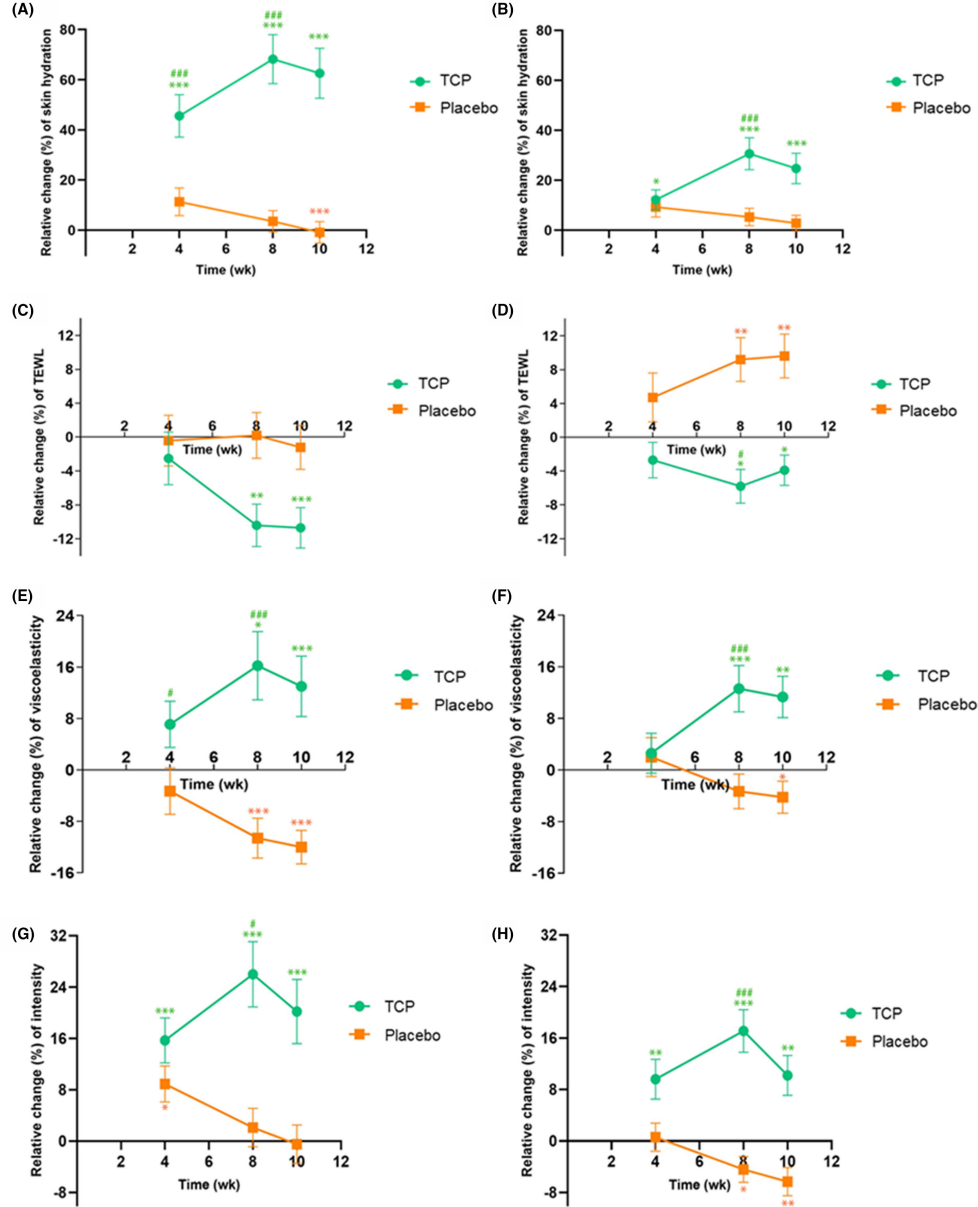
Relative mean change (%) from baseline of the skin hydration in the face (A) and forearm (B), the TEWL in the face (C) and forearm (D), the VE in the face (E) and forearm (F), the skin intensity in the face (G) and forearm (H) at 4, 8, and 10 weeks of TCP (green, *n* = 36) or placebo (yellow, *n* = 36) ingestion. The measurements were performed in triplicate. Data are presented as the mean ± SEM. The statistical analysis was performed using a two‐way repeated measures ANOVA with Tukey's multiple comparisons test. *, **, *** indicates the significant difference at *p* < 0.05, <0.01, and <0.001, respectively when compared with its initial time. #, ### indicates the significant difference at *p* < 0.05 and <0.001 when compared with the placebo group.

At baseline, facial skin hydration levels of the TCP and placebo groups were 47.6 and 46.4 A.U., respectively, with no significant difference between groups. After the administration of TCP for 4 and 8 weeks, the facial skin hydration significantly increased by 15.2 A.U. (to the value of 62.8 A.U.) and 25.0 A.U. (to the value of 72.6 A.U.) (change over baseline, *p* < 0.05), respectively (Table 2). Conversely, the placebo group exhibited no significant change in skin moisture compared to the baseline (*p* > 0.05). When comparing the TCP group to the placebo group at the same intervals, a significant difference in facial skin hydration was observed at 4 and 8 weeks (*p* < 0.05). The relative mean change in facial skin hydration has been calculated and illustrated in Figure 4A. The TCP group showed a progressive improvement in skin hydration at 4 and 8 weeks, with a relative mean change of 45.6% and 68.2%, respectively. Meanwhile, the placebo group showed a smaller increase in skin hydration, with a relative mean change of 11.3% and 3.5% at 4 and 8 weeks, respectively (Figure 4A). After the discontinuation of the test samples for 2 weeks (at 10‐week) of both groups, the facial skin hydration levels in the TCP group remained significantly increased from the baseline (*p* < 0.05), whereas the facial skin hydration level in the placebo group significantly decreased (*p* < 0.05).

Similar results were also obtained for the forearm skin hydration. At baseline, the forearm skin hydration levels for the TCP and placebo groups were 26.4 and 23.6 A.U., respectively, with no significant difference between them. As detailed in Table 2, following daily ingestion of TCP, the forearm skin moisture level rose significantly by 2.2 A.U. (to the value of 28.7 A.U.) after 4 weeks and by 6.7 A.U. (to the value of 33.1 A.U.) after 8 weeks from the baseline (*p* < 0.05). In the meantime, the placebo group exhibited no significant change in its skin moisture level compared to its baseline (*p* > 0.05). The TCP group demonstrated a significant difference in forearm skin hydration compared to the placebo group at 8 weeks (*p* < 0.05). Similar to the facial area, the relative mean change in the skin hydration for the forearm was calculated and presented in Figure 4B. The TCP group showed a trend of increasing the relative mean change (%) in skin hydration from baseline by 12.2% and 30.6% in 4 and 8 weeks, respectively. This was significantly different from the placebo group, which had smaller changes in skin hydration levels, noted 9.3% and 5.3% at 4 and 8 weeks, respectively, as displayed in Figure 4B. After discontinuing the ingestion of test samples for 2 weeks (at 10‐week), the skin hydration level in the TCP group significantly increased from the baseline (*p* < 0.05), whereas no change was noticeable in the placebo group (*p* > 0.05).

The findings suggest that TCP significantly increased skin hydration when administered for 4 and 8 weeks. This effect persists for 2 weeks even after discontinuation of ingestion, indicating that oral ingestion of collagen peptides is efficiently absorbed into the body as amino acids, dipeptides, and tripeptides.^17^ Hydroxyproline‐containing peptides are easily absorbed into the bloodstream and can remain there for a long time. Prolyl‐hydroxyproline and hydroxyprolyl‐glycine are specific amino acids found in collagen and are not sourced from commonly ingested proteins. These hydroxyproline‐rich peptides may stimulate the growth of skin fibroblasts and boost hyaluronic acid production, indicating that they are the active components of dietary supplements that improve SC hydration and skin elasticity.^17^ According to the composition of TCP, it contains a high ratio of di and tripeptides (more than 20% of the peptide content) with glycine 16.20%, proline 17.61%, and hydroxyproline 7.75% (Table S1). These mentioned amino acids are responsible for collagen synthesis and contribute to improved skin properties.^18^ Several studies have shown that the intake of oligopeptide from natural collagen can be digested into amino acids, dipeptides, and tripeptides. The presence of these very tiny molecules facilitated absorption by being directly absorbed into the bloodstream, delivered, and accumulated in the skin.^2^, ^3^ The subsequent effects then occurred on the skin, such as enhancing skin hydration, as found in the study of Bolke et al., measured by skin corneometry.^11^ The moisture content and barrier function of the skin largely depend on the integrity and compositions of SC. Natural moisturizing factor (NMF) is one group of water‐soluble molecules in the SC that regulate skin hydration by attracting and holding water molecules.^19^ Amino acids, the major components of NMF, are generated from the breakdown of filaggrin, a protein found in skin cells.^20^ Collagen peptide intake restored hyaluronic acid synthase and induced filaggrin production.^4^ Filaggrin increases the amino acid contents, which are the main components of NMF in the SC. Therefore, skin moisture levels increased. The findings align with those from a recent randomized, placebo‐controlled trial by Kim et al. In this study, daily oral administration of 1000 mg of low‐molecular‐weight collagen peptide, with a tripeptide (Gly‐X‐Y) content >15%, including 3% Gly‐Pro‐Hyp, resulted in enhanced skin hydration compared to the placebo group after 6 and 12 weeks.^21^ Such findings indicated the potential of low molecular weight of collagen peptides in enhancing skin moisturization and implied that specific amino acids such as glycine, proline, and hydroxyproline might strongly support the effects.

### 3.4 Effect on TEWL

At baseline, there was no significant difference in the TEWL between TCP and placebo groups for both the face and forearm, as indicated in Table 2. After 8 weeks of daily oral administration of TCP, the TEWL values in both the face and the forearm at 8 weeks were significantly decreased from the baseline (*p* < 0.05). In contrast, the placebo group did not exhibit any significant change in TEWL values for the face (*p* > 0.05) but showed a significant increase for the forearm (*p* < 0.05) compared to the baseline. Considering the difference between the two groups at the same time point, it was found that the TCP group showed a significantly lower TEWL value compared to the placebo group at 8 weeks in the forearm (*p* < 0.05). However, no significant difference was observed between the two groups in the face across all measured time points (*p* > 0.05). A comparable outcome was observed in the recent study by Lee et al., where the oral intake of fish‐derived collagen peptide, enriched with dipeptides, Gly‐Pro and Pro‐Hyp, over 12 weeks, exhibited a tend towards a greater improvement in TEWL compared to the placebo group. Nevertheless, the observed difference between the groups did not reach statistical significance.^22^ When considering the relative mean change (%) in the TEWL from baseline, the TCP group displayed a trend in reducing TEWL by −2.5% and −10.4% in the face and by −2.7% and −5.8% in the forearm at 4 and 8 weeks, respectively. In contrast, the TEWL of the placebo group in the face remained consistent, while the forearm showed an increasing trend, as depicted in Figure 4C,D, respectively. The effect of reduction in TEWL persisted even after discontinuing ingestion for 2 weeks, as indicated by the lower value of TEWL of the TCP group compared to the baseline (*p* < 0.05). As known, SC is a skin barrier that prevents water loss. The role of NMF within corneocytes and the importance of the SC intercellular lipid organization (lamellar structure) form a barrier to TEWL. A decrease in TEWL value or a lower percentage of relative mean changes in TEWL indicates more effective prevention of skin water loss. These findings suggest that oral ingestion of TCP may reduce TEWL. This is because the collagen di or tripeptides could potentially boost the filaggrin expression, which is important for SC barrier function.^23^

### 3.5 Effect on skin elasticity

At baseline, the skin elasticity, measured as VE value, showed no significant difference between the TCP and placebo groups for both the face and forearm positions (*p* > 0.05) (Table 2). After 8 weeks, the VE in the face of the TCP group significantly increased from 4.8 MPa at baseline to 5.2 MPa (*p* < 0.05), whereas the placebo group showed a significant decrease in VE from 4.6 MPa at baseline to 3.9 MPa (*p* < 0.05). Similar results were also obtained in the forearm with a significant increase in VE at 8 weeks of the TCP group (*p* < 0.05), whereas the placebo group remained relatively unchanged from the baseline (*p* > 0.05) (Table 2). Compared to the placebo group, the TCP group demonstrated a significant VE at 4 and 8 weeks in the face (*p* < 0.05) and at 8 weeks in the forearm (*p* < 0.05). The relative mean change (%) in VE from baseline at 8 weeks showed an increase of 16.2% for the TCP group in the face, while the placebo group experienced a 10.6% decrease, as shown in Figure 4E. A similar result was found in the forearm. The relative mean change (%) in VE from baseline at 8 weeks of the TCP group tended to be increased by 12.6%. Meanwhile, the relative mean change (%) in VE from baseline at 8 weeks of the placebo group remained unchanged (−3.3%) (Figure 4F). The results are in agreement with the clinical study conducted by Kim et al., which showed that low‐molecular‐weight collagen peptides, derived from the tilapia fish scales having an average molecular weight of less than 1000 Da, significantly increased skin elasticity compared to the baseline and placebo group after being taken for 12 weeks.^24^ This finding reinforces the effectiveness of low‐molecular‐weight collagen peptides, particularly those consisting of di and tripeptides, in enhancing skin elasticity. In addition, skin elasticity is closely linked to the skin's moisture content, especially the SC.^25^ In general, hydrated skin is more flexible and resilient than dry skin. When the skin is adequately moisturized, it becomes plumper and more resilient, thus improving its elasticity.^26^ Taken into account, collagen helps increase the number of fibroblasts and extracellular matrix proteins while decreasing metalloproteinase levels. Oral collagen peptides act in two ways in the dermis: they supply amino acids essential for the building blocks of new collagen and elastin fibers, and they bind to fibroblast receptors, stimulating the production of new collagen, elastin, and hyaluronic acid. These functions can enhance skin elasticity and slow skin aging. In addition, after 2 weeks of discontinuing ingestion, the TCP group showed higher VE in both the face and forearm (*p* < 0.05) compared to their baseline values. This suggests that the oral administration of collagen peptides has a sustained effect on skin elasticity.

### 3.6 Effect on skin density

**FIGURE 5:**
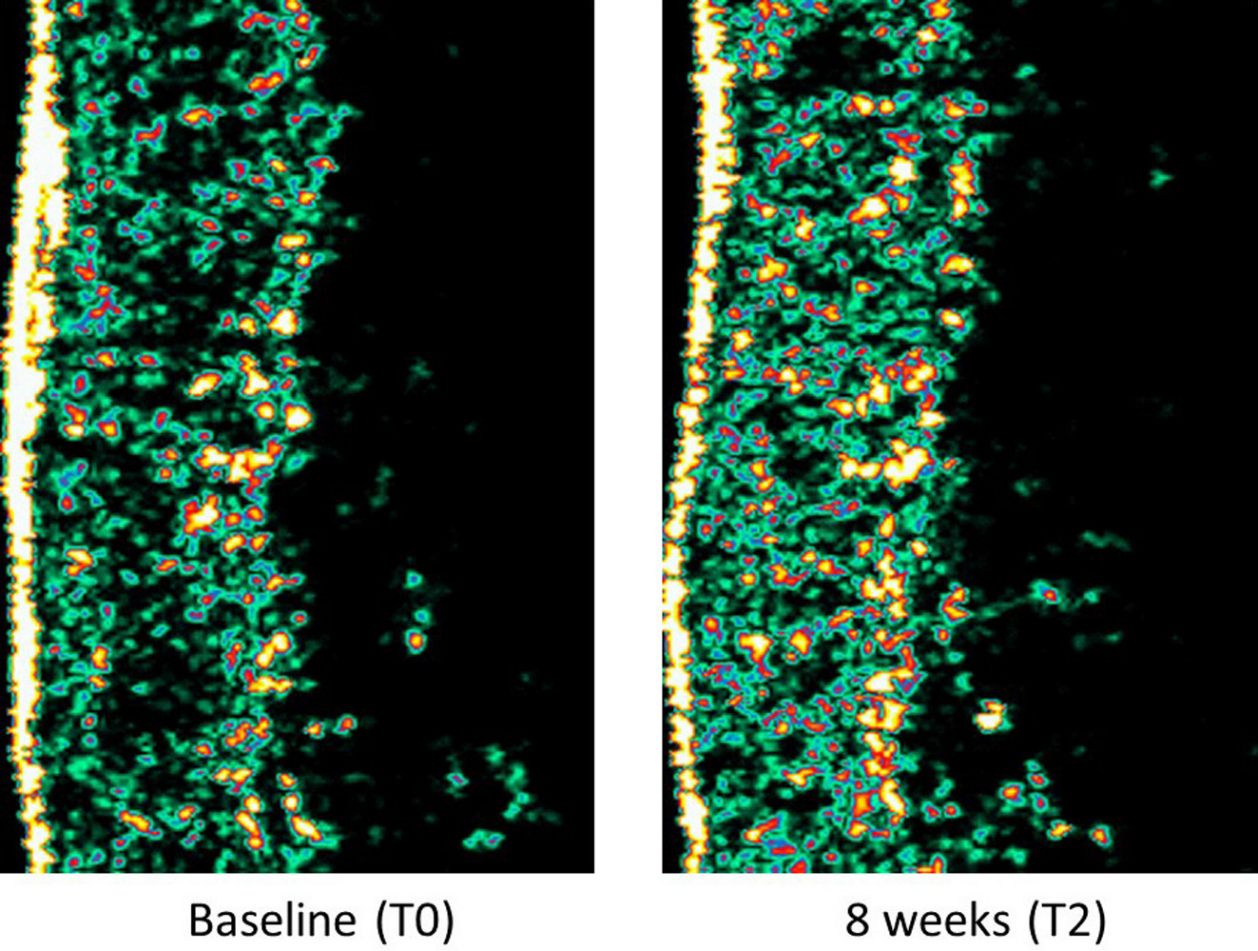
The high‐frequency ultrasonography images of the cross‐sectional view of the skin at baseline (T0) and after ingestion of TCP for 8 weeks (T2). Images were obtained using Dermalab^®^ Combo, 20 MHz ultrasound probe (Cortex Technology ApS, Denmark). Colors represent the intensity of the reflected signal from the skin. Different colors in the image correspond to the varying strengths of the ultrasound signal reflection. Dark colors (e.g., fat layer) indicate low levels of reflection, while light colors (i.e., yellow and green) represent stronger reflections. The dermis layer, visualized with a mixture of colors, reflects the diverse tissue compositions within this layer.

Initially, there was no significant difference in intensity between the TCP and placebo groups in the face and forearm (*p* > 0.05) (Table 2). In the TCP group, the facial intensity increased significantly from 44.4 A.U. at baseline to 50.2 and 53.6 A.U. after 4 and 8 weeks, respectively (*p <* 0.05). Meanwhile, the placebo group showed no significant change in intensity (47.1 A.U. at baseline and 47.0 A.U. at 8 weeks) (*p* > 0.05). At 8 weeks, the TCP group had significantly higher skin intensity than the placebo group (*p* < 0.05). Considering the relative mean change (%) in intensity, the TCP group showed a trend of increase in the relative mean change (%) in intensity from baseline by 15.7% and 26.0% at 4 and 8 weeks, respectively. On the other hand, the placebo group tended to be reduced by −8.9% and − 2.1% over the same periods (Figure 4G). Similar results were observed in the forearm. The intensity of the TCP group in the forearm significantly increased from 44.0 A.U. at baseline to 47.2 and 50.2 A.U. after 4 and 8 weeks, respectively (*p* < 0.05). Meanwhile, the intensity of the placebo group significantly diminished from 45.1 A.U. at baseline to 42.7 A.U. at 8 weeks (*p* < 0.05). Likewise, the TCP group showed a significant difference from the placebo group at 8 weeks (*p* < 0.05). In terms of the relative mean change (%) in intensity, the TCP group presented an increasing trend with rising of 9.6% and 17.1% at 4 and 8 weeks, respectively, whereas the placebo group showed slight reductions of −4.4% at 8 weeks (Figure 4H). Similar findings on skin density were reported by Chang et al. The test product containing hydrolyzed collagen extracted from the striped catfish (*Pangasianodon hypophthalmus*) showed a significant increase in collagen density in the skin, as measured by the ultrasonic evaluation, at both 14 and 28 days compared with baseline and the placebo group.^27^ The examples of high‐frequency ultrasonography images of the skin at baseline and after ingestion of TCP for 8 weeks are shown in Figure 5. Following 8 weeks of TCP intake, the presence of green and yellow parts indicating the availability of collagen and elastic fibers in the epidermal‐dermal layer was more condensed compared to the initial condition. This implied an increased density of skin. Besides, even after a 2‐week discontinuation of the test sample, the TCP group still had the skin intensity higher than the baseline both for the face and forearm (*p* < 0.05).

According to the results above, oral administration of collagen peptides offers enduring benefits for skin density, even post‐treatment cessation. As known, collagen is one of the abundant proteins in human beings that aid in preserving, stabilizing, and strengthening the dermal structures. When consumed orally, TCP can be digested into amino acids and also has more than 20% hydrolysate (di or tripeptides) that can be absorbed through the intestinal mucosa and subsequently transported to the skin. Glycine, proline, and hydroxyproline are amino acids that are associated with the collagen synthesis process and therefore involved with skin health improvement.^18^ TCP supplies the skin with the fundamental components for elastin and collagen production, and it also binds to the fibroblast receptors in the dermis to trigger the formation of elastin and hyaluronic acid. Our findings align with the in vivo study on pigs reported by Matsuda et al., which affirmed that collagen peptide intake strengthens skin mechanical properties by increasing fibroblast density and improving dermal collagen fibril density and diameter.^28^ The effects of oral administration of collagen peptides are pronounced because they act on the deeper layers of the skin, which can reconstruct or improve the structure of the dermis.^29^ This is related to the dermis morphology, as it increases fibroblast density, promotes the formation of collagen fibrils, repairs the damage, and improves skin density. Additionally, collagen in the form of collagen peptides is relatively quickly and extensively absorbed because of the low molecular weights, which allows them to distribute across the tissue efficiently.

While TCP consumption shows positive effects on skin health, further research is needed to fully understand its effects. Our current clinical trial was exclusively conducted on female participants, who are often the primary commercial target for beauty supplements. It is well‐documented that there are physiological differences between male and female skin. These gender‐specific physiological differences may influence the response to TCP. Consequently, exploring the impact of gender on the effectiveness of TCP in skin health is a promising area for future research. Additionally, our current study focused on a specific collagen origin, dose, and exposure time. Exploring the variations in these factors might be a good way to comprehensively understand their relative efficacy. Furthermore, the use of tuna‐derived collagen peptides is relatively new. A deeper understanding of the physiological mechanisms by which TCP promotes skin health is vital for future study.

## 4 CONCLUSION

In summary, TCP showed in vitro efficacy by increasing collagen and elastin synthesis and inhibiting senescence in HDFn primary fibroblasts. The in vitro results were in line with the clinical study. A randomized, placebo‐controlled study confirmed these positive effects on human skin health. Daily consumption of TCP for 8 weeks notably improved skin hydration, reduced TEWL associated with the skin barrier, and enhanced skin elasticity and skin density. The effects were markedly distinct from the placebo group in almost all parameters, with the exception of TEWL in facial skin. Additionally, the effects could be retained for 2 weeks post‐discontinuation. The TCP supplement did not cause any side effects and was safe and well‐tolerated during the entire study period. In addition, the oral administration of TCP allowed the effects to be initiated from the deeper layers of the skin, resulting in long‐lasting improvements to skin health. TCP, sourced from *Katsuwonus pelamis* and *Thunnus albacares*, shows strong potential for development as a dietary supplement.

## AUTHOR CONTRIBUTIONS

Conceptualization: B.M. and V.T.; Methodology and validation: B.M., and V.T.; Investigation and data evaluation: B.M., V.T., A.W., and J.L.; Supervision: B.M and V.T.; Writing and editing of the original draft: B.M. and A.W.; Reviewing and editing B.M., V.T., A.W. and J.L.; and acquisition of funding B.M. All authors have read and agreed to the publication of the manuscript.

## FUNDING INFORMATION

This work is financially supported by the Office of National Higher Education Science Research and Innovation Policy Council through the Program Management Unit for Competitiveness and Thai Union Group PCL. (Grant number C10F650107).

## CONFLICT OF INTEREST STATEMENT

This research paper is supported by the Office of National Higher Education Science Research and Innovation Policy Council through the Program Management Unit for Competitiveness and Thai Union Group PCL. The funding body has no role in the study design, collection, analysis, and interpretation of the data. The funding body also has no role in writing this manuscript. All authors declare that there are no conflicts of interest.

## ETHICS STATEMENT

The study was a randomized, double‐blind, placebo‐controlled clinical trial conducted from April to August 2023 at the Faculty of Pharmacy, Mahidol University. The study protocol was approved by the Institutional Review Board (IRB) of the Faculty of Dentistry/Faculty of Pharmacy, Mahidol University (COA.No.MU‐DT/PY‐IRB 2022/046.1909). The investigation was conducted following the International Conference on Harmonization in Good Clinical Practices (ICH‐CGP) principles and the Declaration of Helsinki. All participants provided written consent for the publication of their anonymized data.

## Supporting information


Data S1.

## Data Availability

The data that support the findings of this study are available from the corresponding author upon reasonable request.

## References

[1] Liu C , Sugita K , Nihei K , Yoneyama K , Tanaka H . Absorption of hydroxyproline‐containing peptides in vascularly perfused rat small intestine in situ. Biosci Biotechnol Biochem. 2009;73:1741‐1747. doi:10.1271/bbb.90050 19661700

[2] Kawaguchi T , Nanbu PN , Kurokawa M . Distribution of prolylhydroxyproline and its metabolites after oral administration in rats. Biol Pharm Bull. 2012;35:422‐427. doi:10.1248/bpb.35.422 22382331

[3] Watanabe‐Kamiyama M , Shimizu M , Kamiyama S , et al. Absorption and effectiveness of orally administered low molecular weight collagen hydrolysate in rats. J Agric Food Chem. 2010;58:835‐841. doi:10.1021/jf9031487 19957932

[4] Jung K , Kim SH , Joo KM , et al. Oral intake of enzymatically decomposed AP collagen peptides improves skin moisture and ceramide and natural moisturizing factor contents in the stratum corneum. Nutrients. 2021;13:4372. doi:10.3390/nu13124372 34959923 PMC8707759

[5] Kang MC , Yumnam S , Kim SY . Oral intake of collagen peptide attenuates ultraviolet B irradiation‐induced skin dehydration *in vivo* by regulating hyaluronic acid synthesis. Int J Mol Sci. 2018;19:3551. doi:10.3390/ijms19113551 30423867 PMC6274925

[6] Campos PM , Melo MO , Calixto LS , Fossa MM . An oral supplementation based on hydrolyzed collagen and vitamins improves skin elasticity and dermis echogenicity: a clinical placebo‐controlled study. Clin Pharmacol Biopharm. 2015;4:142. doi:10.4172/2167-065X.1000142

[7] Sugihara F , Inoue N , Wang X . Clinical effects of ingesting collagen hydrolysate on facial skin properties—a randomized, placebo‐controlled, double‐blind trial. Japanese Pharmacol Ther. 2015;43:67‐70.

[8] Ozcelik M , Kulozik U . The role of maltodextrin concentration in maintaining storage stability of dried fruit foams texturized using plant protein–polysaccharide blends. Food Secur. 2023;12:1673. doi:10.3390/foods12081673 PMC10137890 37107469

[9] Almutairi R , Basson AR , Wearsh P , Cominelli F , Rodriguez‐Palacios A . Validity of food additive maltodextrin as placebo and effects on human gut physiology: systematic review of placebo‐controlled clinical trials. Eur J Nutr. 2022;61(6):2853‐2871. doi:10.1007/s00394-022-02802-5 35230477 PMC9835112

[10] de Miranda RB , Weimer P , Rossi RC . Effects of hydrolyzed collagen supplementation on skin aging: a systematic review and meta‐analysis. Int J Dermatol. 2021;60(12):1449‐1461. doi:10.1111/ijd.15518 33742704

[11] Bolke L , Schlippe G , Gerβ J , Voss W . A collagen supplement improves skin hydration, elasticity, roughness, and density: results of a randomized, placebo‐controlled, blind study. Nutrients. 2019;11:2494. doi:10.3390/nu11102494 31627309 PMC6835901

[12] Lee YI , Lee SG , Jung I , et al. Effect of a topical collagen tripeptide on antiaging and inhibition of glycation of the skin: a pilot study. Int J Mol Sci. 2022;23:1101. doi:10.3390/ijms23031101 35163025 PMC8835374

[13] Offengenden M , Chakrabarti S , Wu J . Chicken collagen hydrolysates differentially mediate anti‐inflammatory activity and type I collagen synthesis on human dermal fibroblasts. Food Sci Human Wellness. 2018;7:138‐147. doi:10.1016/j.fshw.2018.02.002

[14] Shiratsuchi E , Nakaba M , Yamada M . Elastin hydrolysate derived from fish enhances proliferation of human skin fibroblasts and elastin synthesis in human skin fibroblasts and improves the skin conditions. J Sci Food Agric. 2016;96:1672‐1677. doi:10.1002/jsfa.7270 25996804

[15] Edgar S , Hopley B , Genovese L , Sibilla S , Laight D , Shute J . Effects of collagen‐derived bioactive peptides and natural antioxidant compounds on proliferation and matrix protein synthesis by cultured normal human dermal fibroblasts. Sci Rep. 2018;8:10474. doi:10.1038/s41598-018-28492-w 29992983 PMC6041269

[16] Chae M , Bae IH , Lim S , Jung K , Roh J , Kim W . AP collagen peptides prevent cortisol‐induced decrease of collagen type I in human dermal fibroblasts. Int J Mol Sci. 2021;22:4788. doi:10.3390/ijms22094788 33946465 PMC8125628

[17] Nomoto T , Iizaka S . Effect of an oral nutrition supplement containing collagen peptides on stratum corneum hydration and skin elasticity in hospitalized older adults: a multicenter open‐label randomized controlled study. Adv Skin Wound Care. 2020;33:186‐191. doi:10.1097/01.ASW.0000655492.40898.55 32195722 PMC7328867

[18] Albaugh VL , Mukherjee K , Bardul A . Proline precursors and collagen synthesis: biochemical challenges of nutrient supplementation and wound healing. J Nutr. 2017;147:2011‐2017. doi:10.3945/jn.117.256404 28978679 PMC5657141

[19] Maeno K . Direct quantification of natural moisturizing factors in stratum corneum using direct analysis in real time mass spectrometry with inkjet‐printing technique. Sci Rep. 2019;9:17789. doi:10.1038/s41598-019-54454-x 31780805 PMC6882842

[20] Koppes SA , Kemperman P , Tilburg IV , et al. Determination of natural moisturizing factors in the skin: Raman microspectroscopy versus HPLC. Biomarkers. 2017;22:502‐507. doi:10.1080/1354750X.2016.1256428 27805415

[21] Kim DU , Chung HC , Choi J , Sakai Y , Lee BY . Oral intake of low‐molecular‐weight collagen peptide improves hydration, elasticity, and wrinkling in human skin: a randomized, double‐blind, placebo‐controlled study. Nutrients. 2018;10:826. doi:10.3390/nu10070826 29949889 PMC6073484

[22] Lee M , Kim E , Ahn H , Son S , Lee H . Oral intake of collagen peptide NS improves hydration, elasticity, desquamation, and wrinkling in human skin: a randomized, double‐blinded, placebo‐controlled study. Food Funct. 2023;14:3196‐3207. doi:10.1039/d2fo02958h 36916504

[23] Miyanaga M , Uchiyama T , Motoyama A , Ochiai N , Ueda O , Ogo M . Oral supplementation of collagen peptides improves skin hydration by increasing the natural moisturizing factor content in the stratum corneum: a randomized, double‐blind, placebo‐controlled clinical trial. Skin Pharmacol Physiol. 2021;34:115‐127. doi:10.1159/000513988 33774639

[24] Kim J , Lee SG , Lee J , et al. Oral supplementation of low‐molecular‐weight collagen peptides reduces skin wrinkles and improves biophysical properties of skin: a randomized, double‐blinded, placebo‐controlled study. J Med Food. 2022;25:1146‐1154. doi:10.1089/jmf.2022.K.0097 36516059

[25] Sanchez A , Blanco M , Correa B , Perez‐Martin RI , Sotelo CG . Effect of fish collagen hydrolysates on type I collagen mRNA levels of human dermal fibroblast culture. Mar Drugs. 2018;16:144. doi:10.3390/md16050144 29701725 PMC5983275

[26] Dąbrowska AK , Spano F , Derler S , Adlhart C , Spencer NC , Rossi RM . The relationship between skin function, barrier properties, and body‐dependent factors. Skin Res Technol. 2018;24:165‐174. doi:10.1111/srt.1242 29057509

[27] Chang HC , Lin YK , Lin YH , Lin YH , Hu WC , Chiang CF . Hydrolyzed collagen combined with djulis and green caviar improve skin condition: a randomized, placebo‐controlled trial. Curr Res Nutr Food Sci Jour. 2021;9:533‐541. doi:10.12944/CRNFSJ.9.2.16

[28] Matsuda N , Koyama Y , Hosaka Y , et al. Effects of ingestion of collagen peptide on collagen fibrils and glycosaminoglycans in the dermis. J Nutr Sci Vitaminol. 2006;52:211‐215. doi:10.3177/jnsv.52.211 16967766

[29] Tulina D , Beguin A , Pong H , et al. Evaluation of the *in vivo* cosmetic efficacy of the MF3 blue cell serum gel. One‐ and two‐month test results. J Cosmet Dermatol. 2018;17:193‐202. doi:10.1111/jocd.12363 28639749

