# Supplementary material for: The evidence from in vitro primary fibroblasts and a randomized, double‐blind, placebo‐controlled clinical trial of tuna collagen peptides intake on skin health
Source: J Cosmet Dermatol. 2024 Jul 29;23(12):4255–67. doi: 10.1111/jocd.16500 (PMC11626298; doi:10.1111/jocd.16500)

**Supplementary**

**Table S1** The amino acid composition in 100 g of TCP**.**

| Amino acid | TCP (g/100g protein) | |
| --- | --- | --- |
|  |  |  |
| Aspartic acid | | 4.93 |
| Cystine | | Not detected |
| Glutamic acid | | 8.45 |
| Glycine | | 16.20 |
| Histidine | | 2.46 |
| Hydroxylysine | | 0.70 |
| Hydroxyproline | | 7.75 |
| Isoleucine | | 3.52 |
| L-Alanine | | 10.56 |
| L-Arginine | | 7.39 |
| Leucine | | 2.82 |
| Lysine | | 4.23 |
| Methionine | | 1.06 |
| Phenylalanine | | 1.41 |
| Proline | | 17.61 |
| Serine | | 3.17 |
| Threonine | | 3.17 |
| Tyrosine | | 0.70 |
| Valine | | 2.11 |
| Di- & Tri-peptides (%) | | **27.71** |
| Average Molecular Weight by Weight, M.W. (Dalton) | | **1,314** |

**Supplementary S2.** The β-galactosidase staining (blue) of HDFn primary fibroblast indicates cell senescence of control (A), 15.62 (B), 31.12 (C), and 62.50 (D) mg/mL exposure of TCP.


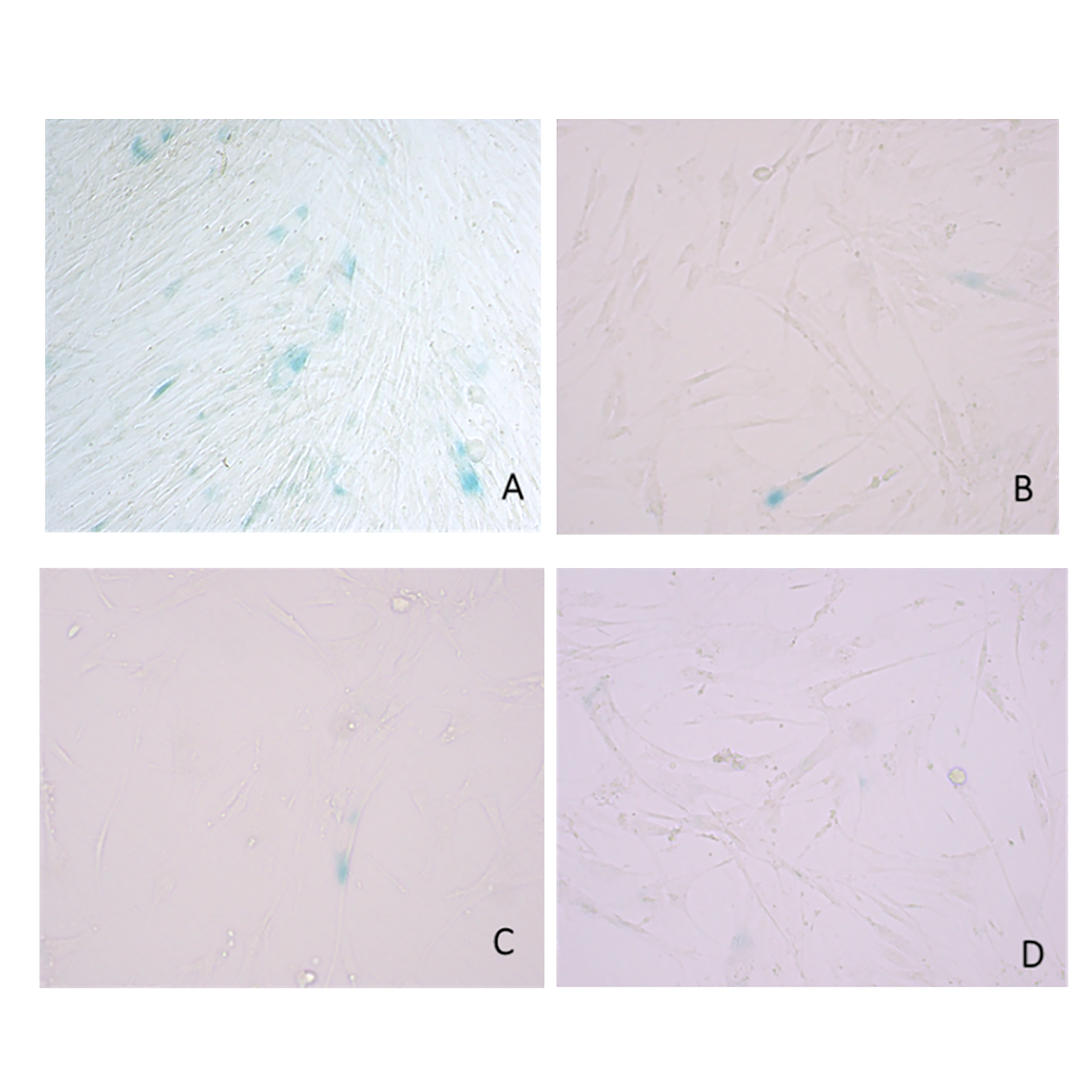

Supplement: Supplementary file 1 — Data S1. [file JOCD-23--s001.docx]
